# Structure and Thermal Stability of Co- and Fe - Intercalated Double Silicene Layers

**DOI:** 10.1186/s11671-017-1874-6

**Published:** 2017-02-10

**Authors:** O. V. Mykhailenko, Yu I. Prylutskyy, I. V. Кomarov, A. V. Strungar

**Affiliations:** 1 Taras Shevchenko National University of Kyiv, Volodymyrska Str., 64, 01601 Kyiv, Ukraine; 2 Vernadsky National Library of Ukraine, Holosiivskyi ave., 3, 03039 Kyiv, Ukraine

**Keywords:** Double-layer silicene, Intercalated Fe and Co atoms, Thermal stability, PM3 and Monte-Carlo simulations

## Abstract

The arrangement of Fe and Co atoms between two silicene planes was theoretically investigated. The research has shown that below 500 K Co atoms form stable lattices—“hexagonal” (with the lattice parameters 0.635 nm of AB configuration) and cubic (with the lattice parameter of 0.244 nm), whereas Fe atoms form cubic lattices only (with the lattice parameter of 0.281 nm). The system intercalated with Co atoms is stable enough at high temperatures up to ~625 K, while the Fe-silicene system is stable only at ~770 K. The silicene UV-spectrum depending on the intercalate concentration and association constant of the “silicene-intercalate” system was calculated.

## Background

Silicene is a newly discovered material that is 1-au layer thin [1–3]. It is a two-dimensional (2D) nanomaterial that is classified as a nanosheet, which has large lateral dimensions up to micrometres, but thickness of several nanometres or less. The unique properties and morphology of such materials make them ideal for a variety of applications, including electronic devices, batteries and sensors. 2D nanosheet of silicene can be considered as an analogue of graphene [3].

Opening a sizable band gap without degrading its high carrier mobility is as vital for silicene to its application as a high-performance field-effect transistor [4].

Besides, the specific surface area of nanomaterials can play one of the key roles on their application. So, the carbon nanotubes (CNTs) distinguish themselves among other nanocarbon structures by a very large specific surface area, the value of which is in the range of 50–1315 m^2^/g [5]. The developed surface area of CNTs reflects on the properties of liquid systems with adding CNTs [6] and by changing the thermodynamic parameters. There is a possibility of enhancing the bioactivity of CNT by carboxyl group functionalization [7]. These examples motivate the use of double-layer silicene as filler in combination with Fe and Co atoms.

Inspired by recent successes in the development of 2D gas-based sensors capable of single gas molecule detection, the adsorption of gas molecules (N_2_, NO, NO_2_, NH_3_, CO, CO_2_, CH_4_, SO_2_ and H_2_S) on silicene nanoribbons (SiNRs) were investigated using the density functional theory (DFT) and nonequilibrium Green’s function (NEGF) methods [8]. The most stable adsorption configurations, adsorption sites, adsorption energies, charge transfer, quantum conductance modulation and electronic properties of gas molecules on SiNRs have been studied. The results indicate that NO, NO_2_ and SO_2_ are chemisorbed on SiNRs via strong covalent bonds, suggesting its potential application for disposable gas sensors.

As the identification of structure-to-property relations is an important task of 2D chemistry and material physics [9, 10], the aims of this work were to study the structure of intercalated Fe and Co atoms double-layer silicene systems under heating by the MM+, PM3 and Monte-Carlo, to calculate the UV-spectrum of silicene layers depending on intercalate concentration as well as to determine the association constant of the “silicene-intercalate” system.

## Methods

Our main object of interest, a double-layer silicene, was modelled with 32 silicon atoms in each plane. The distance between the planes was chosen to be 0.3855 nm (we selected the AB… order of silicon atoms between the planes). Double-layer silicene intercalation is produced via introduction of the intercalate atoms into the interlayer space. As stated above, our goal was to study the system of two silicene planes, with Co or Fe atoms intercalated between them. Of particular interest are the positions of the metal atoms that are relative to each other and the silicon atoms’ mutual positioning of the silicene planes in the presence of the metal intercalates (with or without keeping the AB… order of silicon atoms between the planes), as well as the stability of the system upon thermal treatment [11, 12].

In the model considered, the interaction potential (Leonard-Jones potential) between metal atoms (see Eq. 1) directly mated the pair potential of high energy of atomic excitation [13] and it was described by the Born-Mayer equation within 0–0.68 nm of effective interaction radius (see Eq. 2)

1 $$ U(r)=4\varepsilon \left[{\left(\frac{\sigma}{r}\right)}^{12}-{\left(\frac{\sigma}{r}\right)}^6\right] $$

where *r* is distance between particle centres, *ε* is depth of potential pit, and *σ* is distance at which the interaction energy is equal to zero (parameters *ε* and *σ* characterise atoms of corresponding substances);

2 $$ U(r)= A \exp \left(- r/ b\right) $$

where *A*, *b* is constants, for every pair of colliding particles, and *r* is distance between interacting particles.

To describe the atom interaction at a distance smaller than 0.2 nm, we have used the Tersoff-Brenner potential of interatomic interaction [14]. Total potential energy of system *U* is expressed as a sum of bonding energies for all pairs of atoms forming this system

3 $$ U={\displaystyle \sum_i{\displaystyle \sum_{j> i}\left[{V}_R\left({r}_{i j}\right)-{B}_{i j}^{*}{V}_R\left({r}_{i j}\right)\right]}} $$

where *r*_*ij*_ is distance between *i* and *j* atoms, *V*_*R*_ *(r)* is the exponential function included into the Morse potential type which corresponds to the energies of attraction and repulsion between the atoms, and *B*_*ij*_ * is the function expressing the dependence of binding energy of the *i* and *j* atoms from the angles *θ*_*ijk*_ between the bond *i*–*j* and close bonds *i*–*k* and *j*–*k.*

To describe the atom interaction at a distance greater than 0.21 nm, we have employed the Tersoff-Brenner potential of interatomic interaction [14] along with the Ziegler-Biersack-Litmark pair potential [13]. The length of Si–Si bonds in a silicene layer was 0.220 nm, and the interaction of the Co and Fe atoms with silicene was described by the Lennard-Jones pairwise potential with the energy of 0.112 and 0.110 eV for the Co-silicene and Fe-silicene systems, respectively. The modelled period of one excitation cascade was 2 ps, and the energy conservation law in every calculation cycle was correlated within 0.15%. The initial coordinates of intercalate were selected in conformity with the law of random numbers.

To do the task above, the following numerical modelling scheme was used:

1. the first calculation stage was based on the MM+ molecular mechanics method;
2. the second stage was based on the semi-empirical PM3 method. It should be noted that the main distinction of this method from the others is their different parameterisations. In our case, PM3 method was parameterised to the best match of calculated and experimental molecule formation heats;
3. the third stage was based on the Monte-Carlo method.

To calculate the association constant of the “silicene-intercalate” complex formed, the modified Benes-Hilderbrand method [15] that accounts the data on maximum silicene absorption values at various intercalate concentrations in the UV-spectra was employed.

## Results and Discussion

**Fig. 1:**
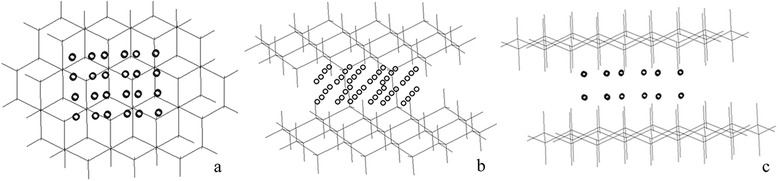
Calculated optimal geometric model of the Co-silicene system: (**a**, **b**) orthogonal projections and (**c**) side view

It was found that at 273 K, Co atoms, when placed between two silicene planes, form a stable quasi-hexagonal lattice with the following lattice parameters 0.635 nm (Fig. 1). At 625 K, however, the hexagonal Co lattice (or the *α*-structure) is transformed into *β*-structure—Co atoms forming a cubic lattice with the lattice parameter of 0.244 nm. The silicene planes do not move relatively each other, and the order of the silicon atoms between the planes remains the same–AB… (akin to the single-crystal silicene). However, when heated above 770 K, the distance between the silicene planes grows to 0.645 nm (AB configuration).

At 273 K, Fe atoms placed between the two silicene planes form a stable cubic lattice with the lattice parameter of 0.281 nm. The silicene planes do not move relatively each other and the order of the silicon atoms between the planes remains the same–AB configuration. The Fe-silicene system turned out to be sufficiently heat resistant in a wide temperature range (up to 770 K) compared to the Co-silicene system (up to only 625 K). Deformational oscillations of the silicene lattice did not exceed 0.014 nm, and those of the Co and Fe lattices, 0.020 and 0.021 nm respectively, suitable for configurational and conformational stability of the system.

**Fig. 2:**
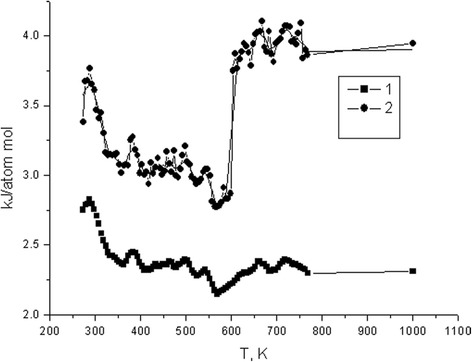
Energy dependence of the Fe-silicene (*1*) and Co-silicene (*2*) systems versus temperature

Apparently, the Co cubic lattice is more sensitive to the temperature changes compared to the Fe lattice. Energy dependence of the Fe- and Co-silicene systems versus temperature is shown in Fig. 2.

One can see that in the range of 273–625 K, the energy of Co-silicene system (Fig. 2, curve 2) gradually decreases, but just above 625 K, it increases sharply. This behaviour is consistent with the transformation of the *α*-structure into the *β*-structure due to the abrupt energy absorption by the system. Further temperature increase causes only gradual disintegration of the cubic Co lattice.

At the same time, the energy of Fe-silicene system (Fig. 2, curve 1) slightly decreases when heated from 273 to ~625 K, and then, with further temperature increase, it reaches a plateau indicating its high resistance to temperature up to ~770 K. When being heated from 273 to 625 K for Co and 273 to 575 K for Fe, the metal lattice becomes somewhat deformed, opposite (relative to the symmetry centre) sites move apart. This transformation is approximately equivalent to the shift of one plane (two atoms) along the main axis of the fourth order by 0.006 nm for Co and by 0.005 nm for Fe.

Increasing the temperature further by ~50–75 K, one can observe greater configurational changes of the Co-silicene system, which correspond to allotrope transitions in Co lattice. At ~625–725 K, a gradual disintegration of the Co lattice is observed. Yet, as the lattice structure breaks down, the interplane distance between the silicene layers remains the same (within the proper range of lattice oscillations). The behaviour of the Fe-silicene system shows certain distinct features.

**Fig. 3:**
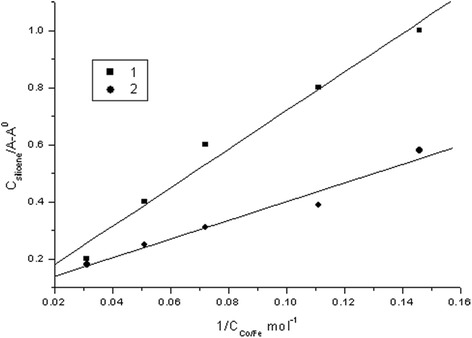
Dependence of silicene absorption with intercalate added in Benes-Hilderbrand coordinates: Fe-silicene system (*1*) and Co-silicene system (*2*)

Finally, theoretical calculations of UV-absorption spectra of the Fe- and Co-silicene systems depending on the intercalate concentration (Fe and Co) in terms of the modified Benes-Hilderbrand method show that the association constant is 6.745 l · mol^-1^ for Fe-silicene system and 3.26 l · mol^-1^ for Co-silicene system (Fig. 3).

## Conclusions

Calculations elucidating arrangement of Co and Fe atoms between two silicene planes have been made. In particular, we demonstrated that at 273 K, Co atoms form a hexagonal lattice, heat resistance up to 625 K. Above this temperature interval, the lattice transforms into a cubic one due to allotropic changes. In the Fe-silicene system, Fe atoms form a cubic lattice, thermally resistant up to 770 K.

Above 725 K for the Co-silicene system and above 770 K for the Fe-silicene system, there is a gradual breakdown of the metal lattices consistent with the order-disorder phase transition. This, in all likelihood, may produce significant changes in the physical and chemical properties of both the intercalated metal and the system as a whole. Concurrently, the distance between the silicene planes was practically unchanged for both Co- and Fe-intercalated systems.

Differences in the behaviour of Co- and Fe-silicene systems stem from the decrease of the atomic radius in the Fe–Co row of the periodic table, which is caused by *d*-compression, i.e., by the increasing attraction of the *ns*-electrons to the nucleus with the increasing charge of the latter. This, in turn, yields a decrease in chemical activity of the metals in the Fe–Co row.

Silicene planes can be considered as inorganic ligands for sandwich structures (similar to ferrocenes). *d*-compression of the electron shells of Co, as compared to Fe, is responsible for the difference in properties of those metals, in particular, the decreasing affinity of Co to aromatic and quasi-aromatic structures (such as silicene). Thus, Co interaction with silicon is weaker than that of Fe. As a result, while intercalated with Co and Fe, the order of silicon atoms between the silicene planes remains the same, i.e., AB configuration.

## Abbreviations

2D: Two-dimensional
CNT: Carbon nanotube
DFT: Density functional theory
NEGF: Nonequilibrium Green’s function
SiNRs: Silicon nanoribbons
UV-spectrum: Ultraviolet-visible spectrum
MM+: Molecular mechanics method
PM3: Parameterised model number 3—semi-empirical method

## References

[1] Aufray B, Kara A, Vizzini S, Oughaddou H, Landri C, Ealet B, Le Lay G (2010). Graphene-like silicon nanoribbons on Ag(110): a possible formation of silicone. Appl Phys Lett.

[2] Lalmi B, Oughaddou H, Enriquez H, Kara A, Vizzini S, Ealet B, Aufray B (2010). Epitaxial growth of a silicene sheet. Appl Phys Lett.

[3] Spencer M.J.S., Morishita T. (Eds.) (2016) Silicene: structure, properties and applications. Springer Series in Materials Science, Springer International Publishing, Switzerland, p 270

[4] Quhe R, Fei R, Liu Q, Zheng J, Li H, Xu C, Ni Z, Wang Y, Yu D, Gao Z, Lu J (2012). Tunable and sizable band gap in silicene by surface adsorption. Sci Rep.

[5] Peigney A, Laurent C, Flahaut E, Bacsa R, Rousset A (2001). Specific surface area of carbon nanotubes and bundles of carbon nanotubes. Carbon.

[6] Korolovych VF, Bulavin LA, Prylutskyy YI, Khrapatiy SV, Tsierkezos NG, Ritter U (2014). Influence of single-walled carbon nanotubes on thermal expansion of water. Int J Thermophys.

[7] Burlaka A, Lukin S, Prylutska S, Remeniak O, Prylutskyy Y, Shuba M, Maksimenko S, Ritter U, Scharff P (2010). Hyperthermic effect of multi-walled carbon nanotubes stimulated with near infrared irradiation for anticancer therapy: in vitro studies. Exp Oncol.

[8] Aghaei SM, Monshi MM, Calizo I (2016). A theoretical study of gas adsorption on silicene nanoribbons and its application in a highly sensitive molecule sensor. RSC Adv.

[9] Radchenko TM, Tatarenko VA, Sagalianov IY, Prylutskyy YI (2014). Effects of nitrogen-doping configurations with vacancies on conductivity in graphene. Phys Lett A.

[10] Radchenko TM, Tatarenko VA, Sagalianov IY, Prylutskyy YI, Szroeder P, Biniak S (2016). On adatomic-configuration-mediated correlation between electrotransport and electrochemical properties of graphene. Carbon.

[11] Mykhailenko OV, Prylutskyy YI, Matsui D, Strzhemechny YM, Le Normand F, Ritter U, Scharff P (2010). Structure and thermal stability of Co- and Fe-intercalated double graphene layers. J Comput Theor Nanosci.

[12] Mykhailenko OV, Prylutskyy YI, Кomarov IV, Strunhar AV (2016). Thermodynamic complexing of monocyclopentadienylferum (II) intercalates with double-walled carbon nanotubes. Nanoscale Res Lett.

[13] Rapaport DC (1995). The art of molecular dynamics simulation.

[14] Tersoff J (1989). Modelling solid–state chemistry: interatomic potentials for multicomponent systems. Phys Rev.

[15] Dorfman S, Mundim KC, Fuks D, Berner A, Ellis DE, Van Humbeeck J (2001). Snapshot of an electron orbital. Mat Sci Eng.

